# Harnessing Engineered Microbial Consortia for Xenobiotic Bioremediation: Integrating Multi-Omics and AI for Next-Generation Wastewater Treatment

**DOI:** 10.3390/jox15040133

**Published:** 2025-08-19

**Authors:** Prabhaharan Renganathan, Lira A. Gaysina, Cipriano García Gutiérrez, Edgar Omar Rueda Puente, Juan Carlos Sainz-Hernández

**Affiliations:** 1 Department of Bioecology and Biological Education, M. Akmullah Bashkir State Pedagogical University, 450000 Ufa, Russia; prabhaharan06@gmail.com (P.R.); lira.gaisina@gmail.com (L.A.G.); 2 All-Russian Research Institute of Phytopathology, 143050 Bolshye Vyazemy, Russia; 3 Centro Interdisciplinario de Investigación para el Desarrollo Integral Regional Unidad Sinaloa, Instituto Politécnico Nacional, Guasave 81049, Mexico; cgarciag@ipn.mx; 4 Departamento de Agricultura y Ganadería, Universidad de Sonora, Blvd. Luis Encinas y Rosales, Hermosillo 83000, Mexico

**Keywords:** microbial consortia, xenobiotic bioremediation, multi-omics integration, environmental DNA, artificial intelligence, antimicrobial resistance genes

## Abstract

The global increase in municipal and industrial wastewater generation has intensified the need for ecologically resilient and technologically advanced treatment systems. Although traditional biological treatment technologies are effective for organic load reduction, they often fail to remove recalcitrant xenobiotics such as pharmaceuticals, synthetic dyes, endocrine disruptors (EDCs), and microplastics (MPs). Engineered microbial consortia offer a promising and sustainable alternative owing to their metabolic flexibility, ecological resilience, and capacity for syntrophic degradation of complex pollutants. This review critically examines emerging strategies for enhancing microbial bioremediation in wastewater treatment systems (WWTS), focusing on co-digestion, biofilm engineering, targeted bioaugmentation, and incorporation of conductive materials to stimulate direct interspecies electron transfer (DIET). This review highlights how multi-omics platforms, including metagenomics, transcriptomics, and metabolomics, enable high-resolution community profiling and pathway reconstructions. The integration of artificial intelligence (AI) and machine learning (ML) algorithms into bioprocess diagnostics facilitates real-time system optimization, predictive modeling of antibiotic resistance gene (ARG) dynamics, and intelligent bioreactor control. Persistent challenges, such as microbial instability, ARG dissemination, reactor fouling, and the absence of region-specific microbial reference databases, are critically analyzed. This review concludes with a translational pathway for the development of next-generation WWTS that integrate synthetic microbial consortia, AI-mediated biosensors, and modular bioreactors within the One Health and Circular Economy framework.

## 1. Introduction

The rapid expansion of global urbanization, industrialization, and population growth has substantially increased wastewater generation. In many low- and middle-income regions (LMICs), inadequate infrastructure results in the discharge of untreated or partially treated effluents into natural water bodies. This threatens aquatic biodiversity, public health, and ecosystem integrity [1,2]. Wastewater treatment systems (WWTS) integrate physical, chemical, and biological treatments to eliminate contaminants, such as organic matter, nutrients, pathogens, and suspended solids. Among these, biological treatment systems, particularly activated sludge systems, are widely used in municipal and industrial WWTS owing to their cost-effectiveness, low energy requirement, and environmental compatibility [3]. Activated sludge systems utilize microorganisms to facilitate organic matter degradation, nutrient recycling, and sludge stabilization [2,4]. Although bacteria are the dominant functional group, archaea, protozoa, fungi, algae, and bacteriophages also contribute to nutrient flux stability and process efficiency [2,5]. Recent evidence highlights the indispensable role of fungi, such as *Phanerochaete chrysosporium*, *Trametes versicolor*, and filamentous *Aspergillus* and *Penicillium* in degrading persistent xenobiotics via extracellular enzymes such as laccases, manganese peroxidases, and cytochrome P450 monooxygenases [6].

Biological WWTS face increasing challenges from recalcitrant xenobiotics, such as pharmaceutical drugs, endocrine-disrupting compounds (EDCs), synthetic dyes, microplastics (MPs), and antibiotic resistance genes (ARGs). These pollutants are often inadequately removed and contribute to effluent toxicity, environmental pollution, and dissemination of ARGs [7,8]. The poor degradability of many xenobiotic compounds is attributed to their structural complexity and low bioavailability, and the absence of specific catabolic genes or regulatory mechanisms in native microbial communities [9,10]. The expression of xenobiotic-degrading pathways is highly regulated by transcriptional regulators that modulate key metabolic functions [9,10,11]. Recent advances in genetic engineering, integrated with high-throughput omics technologies, have enhanced the identification, characterization, and optimization of these catabolic pathways, thereby enhancing the efficiency of microbial biodegradation of xenobiotic pollutants [10,11]. Metagenomic analyses have revealed that benzoate degradation pathways are central to the transformation of aromatic pollutants. These pathways are supported by functional gene clusters for nitrotoluene, aminobenzoate, and polycyclic aromatic hydrocarbon degradation [12]. Under optimal aerobic conditions (pH 7.5, 30 °C, and 0.25% glucose), *Pseudomonas* spp. have been shown to reduce phenolic compounds from 200 mg L^−1^ to ~76 mg L^−1^ within 24 h [13].

Despite the promising potential of biological treatment systems, advanced treatment technologies, such as membrane bioreactors (MBRs) and advanced oxidation processes (AOPs), achieve high removal efficiencies for recalcitrant xenobiotics. However, large-scale applications remain limited by the high energy demands and operational costs, particularly in LMICs [14]. In contrast, nature-based and decentralized solutions, such as constructed wetlands (CWs), anaerobic baffled reactors (ABRs), and septic systems, offer sustainable, low-cost alternatives [15,16]. In particular, CWs have demonstrated high efficacy in the removal of pharmaceutical residues and ARGs. For instance, CW microcosms exposed to antibiotics such as oxytetracycline and enrofloxacin showed >99% removal within three weeks [17]. Similarly, Chen et al. [18] reported antibiotic removal rates ranging from 75.8% to 98.6%, and ARGs are reduced by 64–84% in CWs. Furthermore, oxytetracycline-focused CW systems achieved an antibiotic removal rate of 88.6%, accompanied by a 1000-fold decrease in tetracycline resistance genes (e.g., *tetB* and *tetW*) [19].

Recent advances in high-throughput molecular diagnostics, including metagenomics, environmental DNA (eDNA) profiling, and bioinformatics platforms, such as the Phylogenetic Investigation of Communities by Reconstruction of Unobserved States (PICRUSt) and Functional Annotation of Prokaryotic Taxa (FAPROTAX), have transformed WWT monitoring, facilitating rapid and comprehensive profiling of microbial community composition, ARGs, and pathogenic taxa [20,21,22]. Moreover, they facilitate the elucidation of syntrophic interactions, community succession patterns, and functional compatibility among nitrifiers, methanogens, sulfate reducers, and xenobiotic-degrading heterotrophs [23,24]. In addition, the application of artificial intelligence (AI) and machine learning (ML) algorithms, such as artificial neural networks (ANNs), random forests (RFs), support vector machines (SVMs), and convolutional neural networks (CNNs), has enhanced the predictive modeling efficiency of microbial dynamics and treatment performance. When integrated with multi-omics datasets and environmental parameters, these computational approaches support early warning systems for process instability, ARG dissemination, and xenobiotic persistence [25,26].

This review critically evaluates recent advances in microbial ecology, molecular diagnostics, and bioprocess engineering, with a focus on xenobiotic bioremediation. This study examined the integration of multi-omics platforms with AI-driven biosensing for real-time microbial community profiling and evaluated the structure and functional dynamics of microbial consortia in both aerobic and anaerobic treatment systems. Engineered strategies, such as co-digestion, biofilm-based reactors, direct interspecies electron transfer (DIET) stimulation, and targeted bioaugmentation, are also discussed. Despite these advances, key questions remain as to the extent to which emerging multi-omics and AI tools can overcome persistent limitations in achieving efficient and resilient xenobiotic degradation, and how engineered microbial consortia, comprising bacteria, fungi, and microalgae, can be optimally designed for multi-pollutant wastewater treatment within a circular bioeconomy framework. The novelty of this review lies in its integrative perspective, wherein microbial ecology, multi-omics insights, AI-driven diagnostics, and engineered microbial interactions are synthesized into a holistic model for next-generation xenobiotic bioremediation. In contrast to previous reviews that have addressed these dimensions in isolation, the present work highlights their integration, thereby offering a forward-looking roadmap for the development of scalable, climate-resilient, and One Health-aligned WWTS.

The literature reviewed in this study was identified through a structured search of the Web of Science, Scopus, and Google Scholar databases. Publications from 2010 to 2025 are considered, with priority being given to peer-reviewed journal articles, high-impact conference proceedings, and authoritative reports. The search employed combinations of keywords, such as xenobiotic degradation, microbial consortia, co-digestion, multi-omics, artificial intelligence, bioaugmentation, biofilm reactors, and constructed wetlands. The inclusion criteria are (i) studies reporting original experimental or modeling data; (ii) relevance to wastewater treatment, microbial ecology, or bioprocess engineering; and (iii) quantitative performance metrics for pollutant removal or resource recovery. Studies without experimental evidence, non-peer-reviewed articles, and studies unrelated to environmental biotechnology are excluded. Where possible, the findings from different studies are quantitatively compared to identify performance patterns and trends across treatment strategies.

## 2. Microbial Ecology of WWTS

Microbial-mediated biodegradation of pollutants and nutrient recovery in WWTS vary between aerobic and anaerobic environments. Moreover, they are influenced by several operational parameters, including the influent characteristics, hydraulic retention time (HRT), sludge age, pH, temperature, and reactor configuration [1,27]. In aerobic WWTS, heterotrophic and facultative bacteria utilize dissolved oxygen (DO) to oxidize organic matter into carbon dioxide (CO_2_), water, and microbial biomass. When the influent biochemical oxygen demand (BOD)/chemical oxygen demand (COD) ratio exceeds 0.5, the BOD removal efficiency often reaches 85–95% [28]. Aerobic biodegradation of xenobiotics is predominantly mediated by bacterial taxa such as *Pseudomonas*, *Acinetobacter*, *Bacillus*, and *Flavobacterium*, which metabolize a wide range of pollutants, including plastics, pharmaceuticals, carbon tetrachloride, paracetamol, polystyrene, *n*-alkanes, and polycyclic aromatic hydrocarbons [29,30,31]. These bacteria often form biofilms or bioflocs on pollutant surfaces, such as hydrophobic micropollutants, plastic particles, oily films, or suspended solid organic matter, which provide a supportive structure for microbial attachment. This colonization not only facilitates the physical disintegration of contaminants through localized enzymatic activity but also increases the effective surface area available for microbial interaction, thereby accelerating pollutant breakdown [29,30]. In addition, certain *Pseudomonas* strains exhibit metabolic versatility by simultaneously carrying out heterotrophic nitrification and aerobic denitrification, which significantly contributes to nitrogen removal in WWTS [32]. However, high microbial community diversity does not necessarily correspond to improved degradation efficiency. Functional redundancy, in which many taxa can perform similar functions, confers resilience. Nevertheless, this redundancy can obscure early indicators of process failure, especially when rare but functionally critical specialist taxa are suppressed, thereby reducing long-term performance fidelity [33]. Although enhanced microbial diversity and functional redundancy confer resilience to WWTS, these characteristics can obscure early indicators of functional decline and delay corrective interventions. For instance, in full-scale anaerobic digesters, hydrogenotrophic methanogens sustain methane production when acetoclastic methanogens are inhibited, postponing the detection of ammonia-induced process instability until system failure occurs [34].

In addition to bacterial taxa, fungi, particularly white-rot basidiomycetes and filamentous ascomycetes, possess potent extracellular enzymatic systems that are capable of breaking down aromatic xenobiotics, dyes, pharmaceuticals, and hydrocarbons. These enzymes include laccases, lignin peroxidases (LiPs), manganese peroxidases (MnPs), and cytochrome P450s, which enable non-specific oxidative degradation under diverse environmental conditions [6]. Recent studies have demonstrated that fungal–bacterial consortia outperform single-domain systems in degrading high-strength industrial and agricultural effluents. For instance, a mixed consortium (with bacterial strains including *Kocuria rhizophila*, *Lysinibacillus fusiformis*, *Staphylococcus hominis*, and *Bacillus subtilis*, and fungal strains including *Lichtheimia corymbifera*, *Cladosporium cladosporioides*, *Cladosporium tenuissimum*, *Aspergillus versicolor*, and *Geotrichum candidum*) achieved 93% COD removal from synthetic slaughterhouse wastewater compared to <78% using bacterial consortia alone [35]. Additionally, white-rot fungi such as *T. versicolor* have been shown to produce >2500 U/mg laccase activity while valorizing organic screening in wastewater treatment processes [36]. Nonetheless, incorporating fungi into bioprocesses presents several challenges, including comparatively slower growth than bacteria, potential mycotoxin production, and lower tolerance to predatory pressure issues that are rarely addressed in current bioprocess designs.

Anaerobic digestion (AD) is a highly effective and widely adopted technology for treating high-strength, organic-rich industrial wastewater generated by sugar mills, distilleries, pulp and paper mills, textiles, slaughterhouses, dairy producers, breweries, and the food-processing industry [37,38]. This biological process not only achieves substantial pollutant removal but also generates biogas, including methane, which serves as a renewable energy source [39,40]. Several WWTS that utilize AD have evolved into net energy producers, particularly when the process is integrated with co-digestion strategies and biogas upgrading technologies [39]. The efficacy of AD relies on a syntrophic microbial consortium comprising hydrolytic bacteria, fermentative acidogens, acetogens, and methanogenic archaea that are adapted to diverse industrial substrates. This microbial diversity ensures process stability, functional redundancy, and resilience to operational variation [41,42]. However, redundancy can delay process failure detection; for instance, hydrogenotrophic methanogens may temporarily compensate for inhibited acetoclastic activity, obscuring indications of ammonia toxicity until rapid collapse occurs [33]. Moreover, the implementation of process intensification strategies, such as micro-aeration, physicochemical pretreatments, and integration with other biological processes, including anaerobic ammonium oxidation (anammox) and BESs, can significantly enhance both the pollutant removal efficiency and bioenergy recovery [42,43].

**Figure 1:**
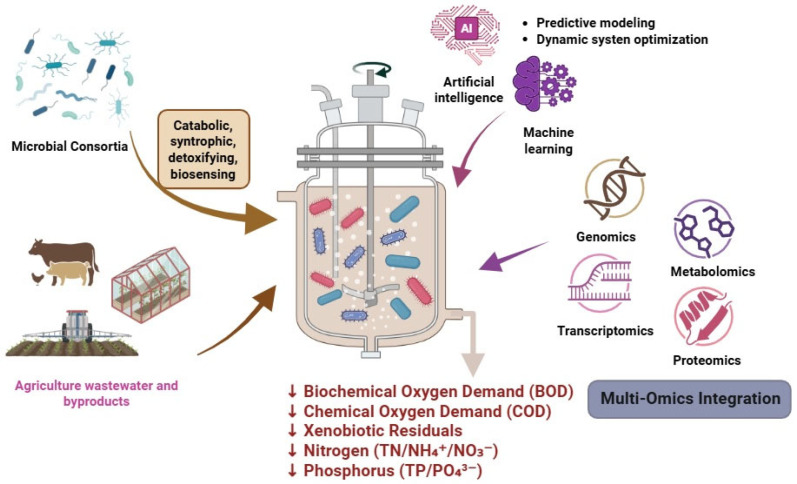
Integrated framework linking engineered microbial consortia, multi-omics analytics, and AI-driven optimization for xenobiotic bioremediation in WWTS. Arrows indicate the directional flow of contaminants into the bioreactor, microbial catabolic and detoxification functions, and the integration of omics data with artificial intelligence for predictive modelling and system optimization.

In metagenomic studies of oxytetracycline-laden wastewater, anaerobic digesters exhibited a 45% reduction in tetracycline resistance genes, with dominant degraders including *Bacteroides* and *Proteiniphilum* [44]. Furthermore, sulfate-rich industrial effluents often utilize sulfate-reducing bacteria (SRB), such as *Desulfovibrio alaskensis* strain 6SR, which can precipitate heavy metals, such as cadmium (Cd), achieving >99.9% Cd removal within 48–144 h [45]. Recent metagenomic surveys in India have confirmed the widespread presence of catabolic genes for chlorobenzene, styrene, atrazine, and naphthalene degradation in both aerobic and anaerobic systems. These pathways are often associated with *Beta*- and *Deltaproteobacteria*, methanogens, and facultative anaerobes, indicating the functional potential of xenobiotic biodegradation under different redox conditions [46]. Recent advances, such as the XenoBug database, which integrates millions of metagenome-derived xenobiotic enzymes, have facilitated the precise selection of microbial strains for the targeted degradation of pesticides, pharmaceuticals, hydrocarbons, and plastics [47], indicating the ecological importance of microbial consortia in adaptive next-generation WWTS. Recent metagenomic insights integrated with predictive functional annotation tools, such as PICRUSt2 and FAPROTAX, have enabled the identification of microbial taxa and gene clusters associated with the degradation of pesticides, hydrocarbons, EDCs, and micropollutants in WWT ecosystems (Table 1 and Figure 1).

## 3. Technologies Enhancing Microbial Bioremediation

Biotechnological interventions significantly enhance the functional performance of microbial consortia in WWTS by improving community stability, ecological resilience, and metabolic efficiency, particularly under xenobiotic stress [5,59]. Strategies such as co-digestion, biofilm engineering, bioaugmentation, and incorporation of conductive materials that promote interspecies interactions, such as DIET, have been increasingly recognized as crucial not only for pollutant removal efficiency and energy recovery but also for creating climate-resilient, adaptable treatment systems capable of functioning under variable socio-environmental contexts [60,61,62,63]. Furthermore, these technologies form the practical application branch of the molecular and AI-based diagnostic strategies explained in Section 4, providing an integrated approach to next-generation wastewater treatment.

### 3.1. Co-Digestion Strategies

Co-digestion involves the simultaneous anaerobic treatment of sewage sludge with organic co-substrates, such as food waste, agricultural residues, and brewery spent grains, to optimize the carbon-to-nitrogen (C/N) ratio, promote microbial diversity, and enhance methane production and xenobiotic degradation by stimulating the functional microbial guilds involved in hydrolysis, acidogenesis, and methanogenesis [64,65]. For instance, co-digestion with food waste has been shown to increase archaeal abundance from 0.4% to 17%, enriching methanogens such as *Methanobrevibacter smithii*, *Methanoculleus receptaculi*, and *Methanospirillum hungatei*, which are resilient to toxicants and have high methanogenic capacities [66]. In contrast, fermentative bacterial phyla, such as *Firmicutes*, *Bacteroidetes*, and *Proteobacteria*, enhance the co-metabolism of recalcitrant organics [67].

In semi-continuous mesophilic systems, methane production increased from 281 to 506 mL CH_4_ g^−1^ vs. with food waste leachate, which was driven by the transition from acetoclastic to hydrogenotrophic methanogenesis [68]. Thermophilic co-digestion systems further exhibit dynamic microbial transitions, in which *Methanothermobacter thermophilus* is replaced by *Methanosarcina thermophila* at high organic loadings, resulting in methane recoveries of up to 98% [69]. Quantitative comparisons from recent studies indicated that co-digestion of food waste and livestock manure under optimized ratios achieved biochemical methane potential (BMP) yields of 0.23 L CH_4_/g^−1^ VS_added_, while integrated kinetic modeling confirmed enhanced resilience against ammonia inhibition [70]. Similarly, simulation-based optimization of a food waste–sewage sludge combination achieved 0.29 L CH_4_ g^−1^ COD removed, with COD and volatile solids (VS) removal efficiencies of 81.5% and 69.2%, respectively [71]. In municipal systems, co-digestion increased methane yields by up to 190% compared to mono-digestion, improving the specific methanogenic activity from 10 to 43 mL CH_4_ g^−1^ vs. [72]. Meta-analyses of more than 400 co-digestion studies have reported average methane yield enhancements of 30–45%, which are largely attributed to enhanced syntropy and redox interactions between fermenters and methanogens [60,67]. These findings highlight the importance of substrate synergy and microbial consortia for achieving high-energy xenobiotic resilience in WWTS. Such findings highlight that co-digestion is not only a methane optimization tool but also a viable strategy for enhancing xenobiotic degradation capacity in diverse WWT settings. While co-digestion generally improves performance, certain feedstocks, particularly lipid-rich substrates, can accumulate long-chain fatty acids (LCFAs), which inhibit methanogenic activity and cause operational issues like foaming and biomass flotation [73]. Similarly, substrates with high free ammonia levels—such as poultry manure—can impair acetoclastic methanogens, reducing methane yield and system stability if ammonia concentrations exceed critical thresholds [74].

### 3.2. Biofilm-Based Systems

Biofilm-based systems are ecologically efficient and can enhance microbial colonization, functional stability, and resistance to toxic compounds, making them suitable for diverse applications, such as wastewater treatment, biocatalysis, and food technology. The surface-attached nature of biofilms allows spatial stratification, forming coexisting aerobic and anaerobic zones that facilitate multistep pollutant transformations under fluctuating operational conditions [75,76,77]. The extracellular polymeric substance (EPS) matrix protects microbial communities from hydraulic stress and toxic insults and contributes to resilience and long-term stability [75,76,78].

Biofilm reactors include Moving Bed Biofilm Reactors (MBBRs), Rotating Biological Contactors (RBCs), and Trickling Filters (TF), which depend on carrier materials such as high-density polyethylene (HDPE), biochar, and polyurethane sponges to facilitate dense and diverse microbial biofilms. Carrier morphology, surface chemistry, and porosity are now being custom-engineered to design microbial community assembly for specific xenobiotic degradation pathways, thus improving system selectivity and efficiency [79,80]. Studies have shown that *Nitrosomonas europaea* and *Nitrosospira multiformis* dominate biofilms in high-temperature industrial reactors, whereas suspended flocs contain *Nitrosomonas oligotropha* and *Nitrosomonas cryotolerans* [81]. Heterotrophic taxa such as *Pseudomonas*, *Flavobacterium*, *Hyphomicrobium*, and *Hydrogenophaga* co-colonize these systems and contribute to co-metabolic xenobiotic degradation via redox coupling and substrate exchange [82,83].

RBCs, which alternate between aerobic and anoxic exposures, enhance sequential nitrification–denitrification processes. The dominant microbial groups include *Thauera*, *Azoarcus*, *Paracoccus*, and *Nitrobacter*, which play vital roles in nitrogen removal and the degradation of nitrogenous xenobiotics. These systems consistently reduce BOD and ammonia (NH_3_) concentrations to <20 mg L^−1^ in the treated effluents [81]. TF, widely used in decentralized and rural settings, support microbial consortia such as *Zoogloea*, *Acidobacteria*, *Sphingomonas*, and *Thiothrix*, which are involved in the degradation of aromatic compounds and the removal of biosurfactants. Under optimized loading, these systems can achieve a BOD of <10 mg L^−1^ and NH_3_ <3 mg N L^−1^ [61]. As biofilms mature, their microbial diversity and metabolic redundancy increase, making them particularly effective in degrading structurally complex pollutants, including dyes, EDCs, and pharmaceuticals. Their adaptability and modularity render biofilm reactors suitable for decentralized WWTS, particularly under variable hydraulic and pollutant loads [76,77]. Although biofilm-based reactors are robust, excessive EPS production may impede mass transfer of nutrients and electron acceptors, leading to internal diffusion limitations [84]. Moreover, biofilm detachment episodes, especially under hydraulic perturbations, can destabilize process performance if not properly managed [85].

#### Biofouling Mitigation in Biofilm-Based Reactors

Biofilm-based systems offer resilience and functional stratification but they are susceptible to biofouling, which impairs mass transfer and long-term stability. Emerging mitigation strategies are now adding practical value:

1. Quorum Quenching (QQ): QQ can significantly mitigate biofouling. A full-scale MBR inoculated with *Acinetobacter guillouiae* ST01 reduced biofilm polysaccharides by 30% and proteins by 47% relative to controls, while halving the fouling rate [86]. In another study, QQ beads introduced into an MBR extended operation time by 2–3×, maintained ~50% QQ activity over 50 days, and reduced polysaccharides and protein by over 40% [87].
2. Functionalized Membranes: PVDF ultrafiltration membranes grafted with quorum-sensing inhibitors (poly(vanillin) brushes) showed a 50% increase in stable flux compared to control membranes during dynamic filtration [88].
3. Anti-fouling Surface Coatings: Superhydrophobic TiO_2_/CuO nanocomposite coatings achieved >6 log_10_ reduction (~99.9%) in *E. coli* and *S. aureus* adhesion within 24 h, meeting ISO 22196 antibiofilm criteria [89].

These examples illustrate that practical biofouling control tools from QQ strategies to anti-fouling coatings offer quantifiable operational benefits. However, their scalability, cost, and compatibility with varied bioreactor configurations warrant careful evaluation, especially for decentralized or resource-limited settings.

### 3.3. Use of Conductive Materials for DIET Stimulation

The addition of conductive materials, such as biochar, magnetite (Fe_3_O_4_), granular activated carbon (GAC), and nanoscale iron oxides, to anaerobic systems has been shown to significantly enhance DIET, which can bypass traditional hydrogen-mediated pathways and improve the degradation of complex or recalcitrant xenobiotic compounds under challenging thermodynamic conditions. These materials act as redox mediators and microbial scaffolds, facilitating electron flow between syntrophic bacteria and methanogenic archaea, thereby accelerating methane production and stabilizing AD processes, particularly under stressors such as high organic loading and temperature fluctuations [90,91,92]. For example, engineered materials such as Fe_3_O_4_-modified biochar have demonstrated increased methane yields by up to 157%, enriched DIET-associated microbial taxa, and improved sludge electrical conductivity [93]. The effectiveness of these materials depends on their conductivity, surface area, and porosity, which influence microbial attachment dynamics and electron transfer efficiency [94,95]. Although DIET is a major mechanism for these improvements, some studies have shown that not all observed benefits can be solely attributed to DIET, as other electron transfer pathways and microbial community shifts also contribute significantly [96,97]. Importantly, the choice, particle size, and dosage of conductive materials require careful optimization, as certain materials (e.g., ferrihydrite and silver nanoparticles) can inhibit AD due to their toxicity or mass transfer limitations [91].

Advanced composites, such as nitrogen-doped Fe_3_O_4_-biochar (Fe_3_O_4_@N-BC), have been shown to upregulate DIET-related genes, including *pilA*, *MmcA*, and *Rnf*, further enhancing the methane yield by 1.75-fold [98]. In high-NH_3_ digesters, nano- Fe_3_O_4_ biochar facilitated syntrophic acetate oxidation (SAO) and increased NH_3_ tolerance, resulting in a 62–139% enhancement in methane production, whereas Fe_3_O_4_ alone increased methane yields by 157%, which was attributed to specific DIET-associated taxa [40,99]. Furthermore, Fe_3_O_4_ supplementation has been shown to enhance DIET-associated taxa, leading to efficient degradation of acetate and benzoate under anaerobic conditions [100,101]. A meta-analysis of diverse study reports indicates that supplementation with Fe_3_O_4_ or biochar typically enhances methane yields by 35–65%, while GAC supplementation consistently improves yields by 25–50% under varied reactor and substrate conditions [102,103]. Moreover, regression analyses show that material surface area correlates strongly (R^2^ ≈ 0.78, *p* <0.01) and electrical conductivity moderately (R^2^ ≈ 0.72, *p* <0.05) with performance gains, underlining the importance of material-specific optimization for scale-up applications [104,105].

Conductive materials also improve system resilience during start-up phase and toxicant stress by increasing microbial adhesion and sludge conductivity by up to 28-fold, thereby promoting process stabilization [106]. Finally, conductive materials not only enhance ecological interactions within anaerobic consortia but also represent a low-cost, scalable augmentation strategy for improving the biodegradation of diverse xenobiotic pollutants. Some conductive amendments (e.g., silver nanoparticles, ferrihydrite) may exhibit toxicity or limited bacterial colonization, which can reduce DIET efficiency or even inhibit anaerobic digestion [100]. Hence, optimization of dosage, material selection, and reactor operational parameters is imperative for reliable scale-up.

### 3.4. Bioaugmentation and Engineered Consortia

Bioaugmentation enhances pollutant degradation by introducing specialized or highly efficient microbial strains into contaminated environments, thereby increasing the degradation of persistent pollutants beyond that achieved by native microbes alone. This approach has proven effective for a diverse range of pollutants, including phenolic compounds, hydrocarbons, pesticides, and polychlorinated biphenyls (PCBs), with degradation rates ranging from 10% to 95%, depending on the pollutant and environmental conditions [107,108,109]. For instance, in dye-laden textile effluents, bioaugmentation with *Bacillus* spp., *Klebsiella pneumoniae*, *Nocardia aminovorans*, *Pseudomonas* spp., and *Shewanella algae* achieved 80–95% azo dye removal through enzymatic and co-metabolic pathways [37,110,111]. In cold climates, bioaugmentation with psychrotolerant or psychrophilic microbial taxa, such as *Aeromonas*, *Acinetobacter*, *Pseudomonas*, and *Klebsiella*, has been shown to significantly enhance the removal of xenobiotic pollutants, such as nitrogen compounds and persistent organics, by 8–9% at 12 °C from wastewater and contaminated soils [62], demonstrating the efficacy of temperature-adapted augmentation strategies for bioremediation.

Engineered synthetic microbial consortia have emerged as promising alternatives to single-strain microbial augmentation for the degradation of complex xenobiotic pollutants in the environment. By formulating multiple specialized strains, these consortia can divide metabolic labor, enabling the breakdown of diverse and recalcitrant compounds such as pesticides, hydrocarbons, plastics, and industrial solvents [112]. The inclusion of fungal partners in such consortia introduces oxidative enzymatic potential that is absent in many bacterial systems. Mycoremediation-based designs using fungi, such as *T. versicolor* and *Aspergillus niger,* contribute to dye, pesticide, and drug degradation through non-specific redox catalysis. When integrated with bacterial hydrolytic pathways, these designs support broader xenobiotic spectra and system resilience [113]. Xu and Jiang [114] developed a multi-strain consortium consisting *Arthrobacter* sp. strain AT5 for atrazine catabolism, *Rhodococcus ruber* YYL for the mineralization of tetrahydrofuran (THF), and yeast–bacterial co-cultures designed for hydrocarbon degradation. These engineered consortia exhibit stable cross-feeding, pH homeostasis, and redox complementarity, which enhance the degradation of complex industrial xenobiotics. Similarly, microalgal–bacterial consortia, such as *Chlorella vulgaris*, *Bacillus licheniformis*, *Scenedesmus obliquus*, and *Pseudomonas fluorescens*, have achieved >95% dye removal and enhanced nutrient recovery, thus offering promise for integrated pollutant and resource recovery [37,110]. These consortia exhibit functional synergy, wherein bacteria degrade complex organic pollutants and release CO_2_ and nutrients, which are subsequently assimilated by microalgae for photosynthetic growth. In return, algae release oxygen that supports bacterial respiration, maintains aerobic conditions, and promotes redox balance. This mutualism enhances the removal efficiencies of dyes, pharmaceuticals, heavy metals, and nitrogenous compounds [1,115]. Furthermore, the biomass generated through these systems can be valorized into biofertilizers, bioplastics, and biofuels, thereby aligning with circular bioeconomic goals [116,117]. These developments represent a transition towards system-level bioaugmentation, incorporating ecological engineering, synthetic biology, and adaptive bioreactor management to address complex and emerging contaminants (ECs) in WWTS. A meta-analysis of 30 recent studies comparing multi-domain consortia (combining bacteria, fungi, and microalgae) with single-domain systems found that engineered multi-domain systems improved pollutant removal efficiency by an average of 25–40% and reduced treatment time by up to 50% [118]. The success of bioaugmentation may be compromised by challenges, such as introduced strains failing to establish due to competition with native microbiota or introduced microbes lacking functional redundancy for sustained performance. Furthermore, substrates rich in ammonia or inhibitory compounds from chicken manure can impede introduced strains unless pre-adaptation or co-augmentation strategies are employed [74].

Although bioaugmentation enhances the degradation of recalcitrant pollutants, it may also shift the native resistome and facilitate horizontal gene transfer (HGT), especially when introduced strains carry mobile genetic elements (MGEs). To mitigate this risk, we recommend resistome screening, plasmid curing, and adaptive laboratory evolution under antibiotic-free conditions prior to deployment. Emerging tools, such as bacteriophage therapy, offer targeted removal of ARG-harboring hosts; for instance, phages have been used to control antibiotic-resistant bacteria in wastewater systems [119,120]. Additionally, CRISPR-Cas-based systems can selectively eliminate resistance genes either by curing plasmids or by inactivating chromosomal ARGs [121]. Deploying these technologies within bioaugmentation workflows supports the development of ARG-conscious engineered consortia that are capable of pollutant removal without exacerbating resistance risks.

In summary, the technological interventions discussed in Section 3, including co-digestion optimization, biofilm reactor innovations, stimulation of DIET, and targeted bioaugmentation, constitute the practical problem-solving characteristic features of advanced WWTS. However, the long-term efficacy of these measures depends on an accurate and timely understanding of the microbial community composition, functional dynamics, and adaptive responses to operational and environmental fluctuations. Such an understanding can be achieved only through the integration of molecular profiling, high-resolution functional diagnostics, and artificial intelligence-assisted predictive analytics, as detailed in Section 4. The synergy between these technological and molecular approaches offers the potential to develop adaptive, self-regulating treatment systems capable of addressing both conventional pollutants and emerging contaminants, while advancing the principles of a circular bioeconomy.

## 4. Molecular Approaches in Microbial Community Profiling

While technological innovations (Section 3) provide applied engineering solutions for enhancing bioreactor performance, their optimization and long-term stability primarily depend on a detailed understanding of the underlying microbial ecology. Modern molecular approaches ranging from environmental DNA (eDNA) analysis and marker gene profiling to whole-genome metagenomics and multi-omics integration offer unprecedented insights into the microbial diversity, metabolic capabilities, and ecological interactions under xenobiotic stress. By enabling precise high-resolution mapping of both taxonomic composition and functional gene expression, these tools can serve as the diagnostic and predictive backbone of next-generation WWTS. The following subsections detail the key molecular strategies that support predictive analytics, facilitate targeted bioaugmentation, and inform adaptive process control in both centralized and decentralized contexts.

### 4.1. Environmental DNA and Marker Gene Profiling

eDNA analysis provides a noninvasive, culture-independent method for characterizing microbial communities in WWTS. High-throughput sequencing of conserved marker genes, particularly the V3–V4 hypervariable region of the bacterial 16S rRNA gene, offers species-level or operational taxonomic unit (OTU) resolution, capturing both dominant and rare taxa with functional relevance [21,23]. eDNA-based amplicon sequencing has revealed temporal and spatial shifts in taxa, such as *Pseudomonas*, Nitrosomonas, *Nitrospira*, *Thiothrix*, and *Candidatus Accumulibacter*, in response to operational variables, such as sludge age, nutrient loading, and reactor design [20,21,23,122]. These taxa are functionally involved in nitrification, denitrification, phosphorus accumulation, and xenobiotic degradation and their abundance is often correlated with effluent quality metrics.

eDNA techniques also allow for the quantification of functional biomarkers, such as ARGs, including *tetB* and *sul1*, thereby directly linking community composition to bioremediation potential and ARG proliferation risk [123]. Dual metabarcoding of bacterial 16S (V3–V4) and eukaryotic 18S (V9) genes enables simultaneous monitoring of protozoa, algae, and fungi, which contributes to sludge stabilization, nutrient assimilation, and microplastic breakdown [20]. Moreover, eDNA profiling has proven effective in detecting transient contamination events, such as the emergence of *Arcobacter* and *Vibrio* in treated effluents, which may indicate system instability, disinfection failure, or pathogen regrowth [21]. Consequently, eDNA-based microbial diagnostics have transformed WWT monitoring from a reactive effluent-focused paradigm to a proactive microbe-informed framework.

Future WWTS must integrate pollutant degradation with ARG containment, involving metagenomic and metatranscriptomic monitoring alongside resistome risk scoring, to detect potential hotspots of HGT in real time [124,125]. AI-driven predictive models can guide operational adjustments, such as the optimization of HRT, adjustment of feed composition, or integration of advanced oxidation process AOPs, to suppress ARG proliferation. Furthermore, synthetic biological interventions are emerging as powerful tools for ARG mitigation. For instance, CRISPR-Cas systems have been demonstrated to selectively eliminate ARGs via plasmid curing or chromosomal targeting [126]. Similarly, engineered bacteriophages, including CRISPR-armed phages, have successfully targeted and reduced ARG-carrying bacterial populations in situ [127]. These elements should be deployed within real-time monitoring intervention loops to dynamically regulate the ARG loads in engineered microbial consortia.

### 4.2. Metagenomics for Taxonomic and Functional Profiling

Shotgun metagenomic sequencing enables comprehensive analysis of microbial taxonomic diversity and functional gene potential, offering a holistic view of bioreactor ecology. This approach allows the recovery of both phylogenetic markers and functional genes involved in biogeochemical cycling and the transformation of xenobiotics in contaminated environments. Key functional genes, such as *amoA* (NH_3_ oxidation), *narG* (nitrate reduction), *mcrA* (methanogenesis), and *atsA* (sulfur assimilation), have been consistently identified in aerobic, anaerobic, and hybrid WWT systems [2,128,129]. Metagenome-guided metabolic flux analysis, in conjunction with stable isotope probing (e.g., ^13^C-labeled bicarbonate), confirms autotrophic carbon fixation by nitrifiers and anammox bacteria, as well as syntrophic methanogenesis by archaea [130]. Recent multi-omics studies have also profiled fungal taxa in WWT systems, linking functional gene expression (e.g., *lac*, *mnp*, and *LiP*) to the degradation of persistent organics. Although often overlooked in bacterial-centric analyses, fungal community profiling via ITS-based metagenomics and metatranscriptomics is increasingly used to track fungal roles in pollutant transformation and enzymatic flux within hybrid systems [113].

Multi-omics integration, including metagenomics, metatranscriptomics, metaproteomics, and metabolomics, facilitates the construction of ecosystem-level functional network models. These networks map microbial taxa to treatment-relevant functions, including nitrification, denitrification, sulfate reduction, and pharmaceutical and dye degradation [128]. Active expression of *amoCAB*, *nirK*, *nirS*, and *nosZ* has been validated under dynamic operational conditions, revealing adaptive metabolic responses to nutrient fluxes and redox variations [2]. Therefore, metagenomic approaches provide a powerful framework for linking microbial identity, function, and ecological interactions, which is vital for rational engineering of stable and efficient consortia in WWT environments.

### 4.3. Functional Inference and Bioinformatics Tools

Bioinformatics tools, such as PICRUSt and FAPROTAX, facilitate the prediction of microbial metabolic potential from 16S rRNA datasets, providing a cost-effective alternative to whole-metagenomic sequencing. The PICRUSt2 tool predicts KEGG orthologs, enzyme commission (EC) numbers, and metabolic pathways, facilitating the identification of nitrifiers, sulfate reducers, xenobiotic degraders, and methanogens in complex microbial consortia [24,131,132]. In contrast, FAPROTAX maps microbial taxa to approximately 90 ecological traits, including anammox bacteria, hydrogenotrophic methanogens, sulfate reducers, and heterotrophic fermenters [24,132].

Function-based diagnostic tools have been applied to NH_3_-oxidizing bacteria, prompting aeration adjustments in MBBRs, whereas a decreased sulfate-reducing potential has led to reactor redesign into two-phase anaerobic systems, improving both sulfate removal and methane yield [131,132]. Thus, functional inference transforms microbial consortium data into diagnostic indicators that promote real-time operational decisions, precision bioaugmentation, and system optimization.

However, a major limitation is the absence of standardized region-specific microbial reference databases, which can lead to biased taxonomic or functional assignments in wastewater treatment plant (WWTP) studies, especially in underrepresented regions. To address this, there is an urgent need for global collaborative initiatives that develop open-access, geo-relevant microbial repositories integrating high-quality genome assemblies, functional annotations, and metadata on operational and environmental parameters [133]. Such repositories should adopt FAIR (Findable, Accessible, Interoperable, Reusable) data principles to enable seamless integration into bioinformatics workflows and to ensure equitable access across research communities. Examples of existing efforts to serve as models include the Earth Microbiome Project, which curates globally sourced microbiome datasets [134], and the Global Water Microbiome Consortium, which focuses on aquatic ecosystems [135]. By extending these frameworks to wastewater-specific contexts, regional microbial diversity can be better captured, thereby improving the resolution and accuracy of microbial community profiling and functional inference in WWTPs. Furthermore, coupling such repositories with accepted metadata standards, such as MIxS, further reduces bias and enhances interoperability [136].

### 4.4. Artificial Intelligence and Predictive Analytics

The incorporation of AI and ML algorithms into microbial ecology has facilitated predictive modeling of community dynamics, ARG dissemination, and treatment outcomes. These tools integrate microbial, physicochemical, and environmental data to simulate system behavior and predict process performance (Table 2).

ANNs trained on multi-omics and environmental variables have achieved R^2^ values between 0.35 and 0.60 for predicting shifts in dominant amplicon sequence variants (ASVs) [25,137]. In MBRs, ANN models link genera, such as *Ferruginibacter* and *Zoogloea*, to biofilm formation, sludge retention time, and fouling risk, achieving a classification accuracy of >91% [138]. SVM, RF, CNN, and ensemble learning algorithms have also been used to associate microbial genera (e.g., *Burkholderiaceae*, *Thauera*, and *Chitinophagales*) with nutrient removal, xenobiotic degradation efficiency, and stability. Furthermore, metaheuristic optimization algorithms, such as particle swarm optimization (PSO), have been used to tune input variables, such as temperature, NH_3_, nitrate, and ARG levels, to optimize microbial performance and effluent quality in near-real time [139]. The integration of field-deployable biosensors, including colorimetric devices for *Escherichia coli*, *Pseudomonas*, and *Enterococcus faecalis*, enables the near-real-time tracking of contamination and operational deviations.

Altogether, the integration of molecular diagnostics with predictive modeling transforms WWTS from reactive systems into proactive intelligence-driven platforms. Nevertheless, as Section 5 elucidates, the translation of these capabilities into widespread applications remains challenged by a number of operational, scientific, and policy-level barriers.

**Table 2:** Integration of AI/ML tools with microbial taxa for predictive application in WWTS.

AI/ML Tool	Target Microbial Taxa	Predictive Output	Application in WWTS	References
Artificial Neural Networks (ANNs)	*Pseudomonas*, *Acinetobacter*, *Sphingomonas*	Abundance of xenobiotic degraders, removal efficiency	Prediction of phenolic and pharmaceutical xenobiotic degradation performance	[25,140]
Convolutional Neural Networks (CNNs)	*Pseudomonas*, *Escherichia coli*, *Enterococcus faecalis*	ARG occurrence and abundance	Near-real-time ARG monitoring for biosafety and co-selection control	[26,141]
Random Forest (RF)	*Pseudomonas*, *Zoogloea*, *Burkholderiaceae*	ARG and heavy metal resistance gene (MRG) trends	Prediction of ARG/MRG proliferation associated with xenobiotic and metal pollution	[142,143]
Support Vector Machine (SVM)	*Thauera*, *Sphingomonas*, *Acinetobacter*	Xenobiotic degradation efficiency	Predicting system response to recalcitrant organics (e.g., PAHs, pharmaceuticals)	[144,145]
Ensemble Learning Models	*Proteobacteria*, *Firmicutes*, *Actinobacteria*	System resilience under xenobiotic stress	Real-time prediction of treatment performance during xenobiotic loading events	[146,147]

## 5. Challenges and Limitations

Despite the tremendous potential of microbial consortia in modern WWT technologies, several critical challenges have hindered their widespread adoption and operational reliability. These limitations arise from biological complexity, ecological variability, reactor-specific constraints, and regulatory gaps.

### 5.1. Immediate Operational Hurdles

#### 5.1.1. Microbial Community Instability and Functional Redundancy

A primary limitation of biological WWTS is the instability of the microbial community composition under fluctuating environmental conditions. Variations in influent composition, pH, temperature, salinity, and HRT can disrupt microbial homeostasis, reducing key functional groups, such as nitrifiers, phosphate-accumulating organisms, and syntrophic consortia. In anaerobic digesters, such variations reduce methanogenic archaea and disrupt metabolic networks, leading to reduced biogas yield. Microbial consortia exhibit functional redundancy, where multiple taxa perform similar metabolic functions, obscuring early indicators of stress and delaying process deterioration detection. For instance, in full-scale anaerobic digesters that treat brewery wastewater, methane production is maintained despite seasonal community turnover [148]. Anammox reactors show high redundancy scores (0.82) among genera such as *Candidatus Brocadia*, *Kuenenia*, *Scalindua*, *Jettenia*, and *Anammoxoglobus*, sustaining nitrogen removal under fluctuating conditions [43].

Saline-activated sludge systems adapt through halotolerant genera without affecting nitrogen removal efficiency [149,150], whereas CWs exhibit turnover with metal-reducing taxa [150]. However, toxicants in textile, petrochemical, tannery, and agrochemical effluents can reduce microbial diversity and eliminate stress-sensitive degraders.

#### 5.1.2. Biofilm Management and Reactor Fouling

Biofilms in WWTS, including MBBRs, Membrane-Aerated Biofilm Reactors, and RBCs, provide advantages, such as microbial protection, surface colonization, and metabolic compartmentalization. However, uncontrolled biofilm development can cause operational issues, such as membrane fouling, clogging, uneven substrate distribution, and reduced reactor performance. In aerobic digesters, overgrown biofilms can develop oxygen-limited inner cores, whereas excessive hydrodynamic shear can slough the biomass and degrade effluent quality. Filamentous bacteria, such as *Microthrix parvicella* and *Nocardia* spp., cause foaming, sludge bulking, and poor settleability, particularly at low temperatures or high fat, oil, and grease (FOG) concentrations [151,152].

Filamentous taxa dominate fouling biofilms in step-aerated MBRs, increasing transmembrane pressure (TMP) and reducing membrane permeability [151]. These bacteria synthesize EPS to form biocakes that are resistant to membrane surfaces. MBRs and MBBR-MBR hybrids treated with saline or pharmaceutical WWTS are susceptible to biofouling by microbial communities, including *Rhodobacter*, *Rhizobium*, *Nitrobacter*, *Ottowia*, *Ferruginibacter*, *Dyella*, and *Clostridium* spp. [151,152,153]. Metagenomic studies have shown that salinity and operational conditions influence biofilm communities, from autotrophic nitrifiers to heterotrophic taxa, thereby affecting system maintenance.

Several strategies have been developed for managing biofilm formation.

1. Operational optimization: Maintaining the filament index (FI) between 1.5 and 3, DO >2.5 mg L^−1^, and food-to-microorganism (F/M) ratios <0.65 g COD g^−1^ MLSS d^−1^ minimized filament growth and stabilized membrane performance. Under these conditions, the increase in TMP remained <2 kPa, whereas deviations can cause an increase in TMP of >14 kPa [151].
2. Chemical control: Low concentrations of oxidants such as hydrogen peroxide, chlorine, or foam suppressants inhibit filamentous organisms. However, such interventions must be cautiously applied to avoid disrupting the beneficial microbial communities [154].
3. Biological control: Quorum-quenching (QQ) bacteria, such as *Pseudomonas putida* and *B. subtilis*, immobilized on microporous carriers disrupt quorum sensing and reduce EPS synthesis and biofilm formation [155].
4. Reactor design: Spatial segregation of nitrifier–denitrifiers minimizes fouling while sustaining nutrient removal.

Effective biofilm management requires an integrative approach that combines microbial ecology, process engineering, and real-time monitoring to control problematic taxa and to ensure process stability and reactor efficiency.

#### 5.1.3. Scale-Up Constraints and Regional Limitations

Although advanced microbial technologies offer transformative potential, the large-scale deployment of low-resource or decentralized WWTS remains challenging. High-throughput sequencing platforms, such as Illumina MiSeq and Oxford Nanopore MinION, are increasingly used in Africa and Southeast Asia for water quality monitoring. However, they require substantial infrastructure, skilled personnel, a reliable power supply, and cold-chain logistics, limiting their feasibility in the Global South. Moreover, many microbial consortia used in pilot-scale trials have been optimized for temperate climates. Species such as *Nitrosomonas*, *Nitrospira*, *Thauera*, *Azoarcus*, *Accumulibacter*, *Tetrasphaera*, and *Candidatus Competibacter* perform poorly under tropical and arid conditions. In contrast, native tropical microbial consortia include *β-Proteobacteria*, *Nitrospirae*, and genera such as *Ottowia* and *Defluviicoccus*, which are climate-adapted, but functionally distinct [142]. For instance, native microalgal–bacterial consortia of *Chlorella*, *Scenedesmus*, and *Pediastrum* with heterotrophs, such as *Pseudomonas*, *Azospirillum*, *Bacillus*, and *Methylobacterium,* have achieved >80% nutrient removal in tropical environments [27]. However, implementation challenges include the absence of standardized regional microbial databases, inconsistent eDNA protocols, and adaptive reactor design. Inconsistencies in sampling methods and the limited accessibility of portable sequencing tools constrain real-time diagnostics in decentralized systems [156].

To address these challenges, regionally adapted native microbial engineering models have been developed to integrate the local biodiversity, climate-resilient taxa, and simplified molecular diagnostic tools. The incorporation of thermotolerant nitrifiers and phototrophic heterotrophic consortia can improve performance at elevated temperatures. The development of geoclimate-specific microbial databases, streamlined eDNA pipelines, and validated bioinformatics workflows will facilitate accurate functional inferences, targeted bioaugmentation, and the scalable implementation of sustainable WWTS under diverse climatic and socioeconomic conditions worldwide.

### 5.2. Fundamental Scientific and Systemic Barriers

#### 5.2.1. Recalcitrant and Emerging Contaminants

Modern WWT effluents contain recalcitrant ECs, such as pharmaceutical residues, personal care products (PPCPs), EDCs, synthetic dyes, and MPs, which resist conventional activated sludge treatment because of the absence of essential enzymatic pathways in the native microbial consortia. Consequently, many ECs that persist in WWTS pose significant ecological and public health concerns. Pharmaceuticals, such as ibuprofen, diclofenac, and carbamazepine, exhibit <50% removal efficiency in conventional systems, requiring specialized microbial degraders for effective bioremediation. Bacterial strains such as *Sphingomonas wittichii*, *Pseudomonas stutzeri*, *Achromobacter*, *Bosea*, *Labrys neptuniae*, and *Bordetella petrii* can achieve up to 99% mineralization of ibuprofen [7]. *Rhodococcus* species degrade non-steroidal anti-inflammatory drugs (NSAIDs) through co-metabolic pathways over days to weeks [157], whereas biofilter systems enriched with *Azospirillum*, *Rhodanobacter*, *Ferrovibrio*, *Sphingopyxis*, and *Prosthecobacter* target phenolic and chloroaromatic compounds. Furthermore, *Pseudomonas* spp., *Acinetobacter*, and *Klebsiella* strains remove EDCs, such as bisphenol A, nonylphenol, and 17β-estradiol, with nearly 100% efficiency [8].

Plastic-degrading microbes such as *Ideonella sakaiensis*, *Aestuariicella hydrocarbonica*, *Parengyodontium album*, *Zalerion maritimum*, and *Aspergillus flavus* degrade polyethylene, polypropylene, and polystyrene using PETase, laccase, peroxidase, cutinase, and depolymerases [158,159]. Additionally, strains such as *Bacillus cereus*, *Bacillus gottheilii*, *Stenotrophomonas maltophilia*, *Rhodococcus*, and *Pseudomonas gessardii* have shown degradation potentials of up to 7.4% for various polymers [159].

Hybrid remediation strategies have been developed to enhance electrocoagulation (EC) removal. These include the following:

1. AOPs, such as ozonation, UV/H_2_O_2_ treatment, and Fenton reactions, chemically oxidize recalcitrant organics.
2. Enzyme-based bioreactors with immobilized laccases and peroxidases from *T. Versicolor* and *Phanerochaete chrysosporium* target phenolics, dyes, and EDCs [7,160].
3. BESs, such as CWs and microbial fuel cell hybrids, integrate microbial and electrochemical degradation for EC breakdown [160].

However, these hybrid systems present operational challenges, including high energy demands, maintenance complexity, toxic byproduct generation, and complex reactor configurations, which require further research. Therefore, next-generation models must integrate engineered EC degraders with catalytic, electrochemical, and physical removal technologies in order to enhance environmental protection.

#### 5.2.2. Antibiotic Resistance Genes and Horizontal Gene Transfer

WWT facilities are critical hotspots for the emergence and dissemination of ARGs, which pose significant threats to ecological integrity and public health. ARGs, such as *bla*, *tet*, *sul1*, *ermB*, *aadA*, and *qnrS*, are detected in both the influent and effluent, indicating incomplete elimination during conventional treatment [22]. ARGs are often associated with mobile genetic elements (MGEs), including plasmids, transposons, integrons, and bacteriophages, which facilitate HGT across diverse taxa [161].

Metagenomic and nanopore-based sequencing have shown that both pathogenic and non-pathogenic bacteria carry and exchange ARGs in WWTS [161,162]. Although primary and secondary treatments reduce ARG loads, there is a resurgence in secondary clarifiers and effluents owing to the accumulation of biomass. For instance, ARGs such as *blaNDM-1*, *sul2*, *ermB*, and *vanA/B/C* persist in treated effluents [163]. HGT in WWTS mainly occurs via conjugation (*IncQ* plasmids and class 1 integrons), transduction (*Siphoviridae* and *Myoviridae*), transformation (extracellular DNA uptake in biofilms), and vesicle-mediated gene exchange, particularly in activated sludge and biofilms, where bacteria such as *Aeromonadaceae*, *Moraxellaceae*, and *Bacteroidetes* create HGT-prone microenvironments [161].

Environmental co-selective agents, including antibiotics, heavy metals, and micropollutants, induce bacterial SOS responses that enhance the mobilization of MGEs and ARGs [164,165]. Textile wastewater contains 3–13 times more MGEs than domestic wastewater, leading to higher downstream ARG transfer [166]. Comparative genomics has shown that multidrug-resistant isolates from WWTS share plasmids with clinical strains carrying more than 200 distinct IS6-family insertion sequences [165]. Moreover, ARGs persist after combined sewer overflow events and inadequate UV/chlorination treatment, thereby posing ecological risks after discharge [164].

#### 5.2.3. Regulatory Gaps, Policy Challenges, and Biosafety Considerations

The lack of standardized regulations governing bioaugmentation agents, genetically modified organisms (GMOs), and ARG monitoring remains a critical barrier to innovation. Regulatory inefficiencies particularly affect synthetic microbial consortia and gene-edited strains, which lack guidelines for environmental release, containment, long-term monitoring, and biosafety assessment. Emerging technologies, such as CRISPR-engineered microbial strains for pollutant degradation and synthetic consortia for enhanced nutrient removal, operate without comprehensive policy frameworks to assess their ecological impact [167,168]. The United States follows a Coordinated Framework involving the FDA, USDA, and EPA; the European Union has implemented GMO Directives 2001/18/EC and 2009/41/EC; and India regulates biosafety under the Environment Protection Act, 1986, through the DBT and GEAC [169,170].

WWTS are a high-risk environment for ARG propagation through HGT. Studies have shown that bacteria such as *Acinetobacter*, *Enterobacter*, *Sphingomonas*, *Bacteroides*, and *E. coli* exchange plasmid-borne ARGs, including *bla_TEM_*, *sul1*, *qnrS*, *tetM*, and *mcr-1*, within WWTS, thereby spreading resistance downstream [168,171]. Extracellular ARGs, such as *nptII* and *bla*, from transgenic crops or biowastes, remain intact in anaerobic digestates and are transferred to soil bacteria [172]. However, a major regulatory gap exists in the absence of a defined ARG discharge threshold. Although EU Regulation 2020/741 requires *E. coli* monitoring in treated water for agricultural reuse, it lacks ARG limits, although resistance markers for sulfonamides, quinolones, macrolides, and tetracyclines have been consistently detected in municipal and industrial effluents [168,172].

Therefore, a science-based risk assessment framework is urgently required to incorporate HGT kinetics, ARG persistence, and high-risk pathogens, such as ESBL-producing *E. coli*, *Acinetobacter* spp., and *Enterobacter* spp. This should also account for environmental co-selectors such as heavy metals, textile dyes, and pharmaceutical residues, which promote ARG proliferation in WWTS [90,133]. Finally, standardized global biosafety classifications, containment guidelines, discharge thresholds, monitoring protocols, and emergency response mechanisms are proposed to facilitate innovation in microbial wastewater biotechnology. Insights from the Cartagena Protocol, EU GMO Directives, and India’s DBT/GEAC framework offer valuable guidance for formulating potential context-specific biosafety regulations within the wastewater sector.

## 6. Future Perspectives: Towards Precision Microbial WWTS

The future of WWTS relies on leveraging microbial consortia not only as passive degraders but also as programmable, adaptive biological systems capable of responding to complex, variable, and emerging wastewater contaminants. Achieving this goal requires an integrated approach to synthesize microbial ecology, synthetic biology, bioreactor engineering, and AI-enabled control systems. This section outlines five strategic frontiers for the development of precision biotreatment platforms that align with sustainability, resilience, and circular economic imperatives.

### 6.1. Engineering Resilient Microbial Consortia

Rational design and optimization of synthetic and semi-synthetic microbial consortia have emerged as promising strategies to address treatment inefficiencies in high-strength industrial and agrochemical effluents. Recent advances have focused on the development of engineered consortia with synergistic metabolic function. By integrating hydrolytic bacteria to decompose complex polymers, denitrifying genera to remove nitrogen, methanogenic archaea for methane production, and xenobiotic degraders to enhance the removal of antibiotics, pesticides, and phenolic compounds, these engineered consortia exhibited synergistic metabolic complementarity and ecological robustness. [114,173,174]. For instance, consortia comprising hydrolytic bacteria, denitrifiers, and methanogens, such as *B. subtilis*, *Thauera* spp., and *Methanosarcina barkeri*, have demonstrated >85% COD removal and methane yields exceeding 300 mL CH_4_ g^−1^ vs. under mesophilic conditions [173]. Synthetic biology tools, including CRISPR–Cas gene editing and modular gene circuits, enable the precise tuning of stress tolerance, redox pathways, and substrate utilization profiles in key microbial chassis, such as *P. putida* and *B. subtilis* [175].

Multi-omics optimization and metabolic network modeling further enhance the predictability and performance of such consortia under fluctuating environmental conditions. Cross-feeding dynamics, metabolic buffering, and ecological redundancy contribute to functional stability. [173,174]. Furthermore, halotolerant, thermotolerant, and phototrophic–heterotrophic combinations are promising for decentralized systems operating under extreme or variable climates [176]. Thus, these engineered consortia represent modular and scalable platforms for next-generation resilient and energy-efficient wastewater ecosystems.

### 6.2. Scaling up Bioelectrochemical and Energy-Positive Systems

The integration of AD with BESs, such as microbial fuel cells (MFCs) and microbial electrolysis cells (MECs), offers the dual advantage of sustainable bioremediation and energy recovery. These systems rely on electrogenic microbial biofilms such as those formed by *Geobacter* spp. and *Methanosarcina*, which facilitate extracellular electron transfer through conductive pili and redox-active compounds. [177,178]. In pilot-scale MEC–AD hybrids, the addition of Fe_3_O_4_ nanoparticles, carbon felt, or GAC has been shown to increase methane production by up to 49%, along with COD removal rates of >90% [179,180]. Carbon-based electrode materials also enhance microbial attachment, promote DIET, and improve methanogenic activity. For instance, GAC-based cathodes achieve methane fluxes of up to 65 L CH_4_ m^−2^·d^−1^, which is 3.8 times higher than those of unamended controls [181].

Scaling innovations include brush-type, tubular, and stainless steel felt electrodes, which support higher biomass loading and biofilm stability [182,183]. However, operational challenges such as electrode fouling, inconsistent biofilm formation, and material degradation require engineering solutions [182]. Real-time biosensor integration, antifouling coatings, and high-surface-area electrode designs are essential for transitioning BESs from laboratory prototypes to field-ready decentralized WWTS, which are net energy generators rather than energy consumers [183].

### 6.3. Integration of Artificial Intelligence and Smart Diagnostics

AI is poised to revolutionize microbial wastewater engineering by enabling real-time decision-making, predictive control, and intelligent bioprocess optimization. AI/ML algorithms, including ANNs, RFs, SVMs, and PSO, have been effectively applied to predict nitrification performance, sludge bulking events, and ARG emergence based on multi-omics and physicochemical parameters [25,184]. For instance, ANN models trained on microbial and operational inputs (e.g., ammonium, nitrate, DO, and microbial abundance) have achieved R^2^ values >0.6 for predicting post-shock nitrifier recovery and guiding aeration adjustments [25,185].

AI-powered “soft sensors” that combine MFC signals with TSS, ORP, and DO data can predict microbial blooms, reactor instability, ARG emergence, and treatment inefficiencies, and they facilitate dynamic operations [185,186]. The integration of BES sensors with ANN-guided biological nutrient removal (BNR) models facilitates the prediction of influent carbon uptake patterns and the optimization of treatment performance [25,185]. These innovations support closed-loop control, wherein the system outputs dynamically inform process inputs, thereby leading to adaptive and self-regulating WWTS. The integration of AI, omics, IoT, and microbial ecology represents a paradigm shift towards digital bioreactors and microbial cyber–physical systems.

### 6.4. Microbial Standardization and Regionalization

A major barrier to field-scale implementation is the lack of standardized microbial diagnostics and region-specific microbial reference datasets.

First, standardized molecular workflows are essential for reproducibility and comparability across studies and treatment systems, including standardized protocols for eDNA sampling, DNA/RNA coextraction, 16S/18S rRNA gene amplification, and metagenomic analysis. Although platforms such as Illumina MiSeq/HiSeq, PacBio, and Oxford Nanopore are widely used, variations in sequencing depth, hypervariable region selection, and bioinformatic processing yield inconsistent microbial profiles. For example, a meta-omics analysis of aerobic granular sludge showed that standardized DNA extraction methods and bioinformatics tools, such as Fastp, VSEARCH, and GTDB, significantly improve taxonomic reliability [4]. Comparative studies of swine wastewater systems have shown methodological biases in the selection of the 16S rRNA hypervariable region, indicating the importance of standardized region selection and reference database alignment [187].

Second, establishing geographically stratified microbial reference databases is crucial for interpreting the microbial consortium dynamics under different climatic regimes. For instance, tropical WWTS are often enriched with specific taxa, such as *Nitrosomonas*, *Nitrosospira*, *Nitrospira*, *Thauera*, *Azoarcus*, *C. Accumulibacter* and *Tetrasphaera*, whereas temperate systems contain species such as *Arcobacter*, *Pseudomonas*, *Cloacibacterium*, *Acinetobacter*, *Polaromonas*, and psychrophilic species (*Mycobacterium* and *Polaromonas*) [59,142].

Third, the development of open-access platforms that correlate microbial profiles with treatment performance is essential for real-time optimization. Tools such as COMETS and FAPROTAX have identified nitrogen-transforming taxa in various sludge types, thereby providing insights into functional redundancy and pathway activity [2]. Integrating these tools into centralized open-source platforms facilitates predictive region-specific control strategies, enhances microbial risk prediction, targeted bioaugmentation, real-time diagnostics, and regulatory cohesion, and enables international benchmarking between South Asian and sub-Saharan African treatment models. Ultimately, microbial standardization and regionalization are crucial for adapting WWT technologies to specific contexts and promoting equitable access to sustainable solutions in tropical, arid, and peri-urban zones.

### 6.5. Policy Innovation and Biosecurity Models

Scaling advanced engineered microbial consortia, such as *E. coli* and *Synechococcus elongatus* for biofuel production, *Geobacter* and *Methanosaeta* systems for methane enhancement through DIET, and engineered *AcidithioBacillus ferrooxidans* strains for bioleaching, requires efficient multilayered biosecurity frameworks to mitigate ecological and human health risks [5,188,189]. Risk assessment protocols must address potential HGT, environmental persistence, synthetic trait dissemination, and mobilization of ARGs, particularly in consortia containing *P. putida*, *Acinetobacter*, *B. subtilis*, and *Clostridium* spp., which are often used for bioremediation [5,188,190]. Therefore, legal clarity regarding defined biosafety tiers is essential for classifying synthetic constructs, including cyanobacterial heterotrophic strains, thermoacidophilic metal-precipitating strains, and fungal–cyanobacterial consortia, under specified containment levels, environmental release protocols, and quantitative disinfection benchmarks (e.g., ≥5-log ARG reduction). These frameworks must incorporate ARG monitoring when deploying consortia with taxa, such as *Acinetobacter*, *Streptomyces*, *Aspergillus*, and *Enterobacter* spp. Emerging data suggest that microalgal–bacterial systems, such as *C. vulgaris* and *Bacillus licheniformis*, can reduce extracellular ARGs. However, its validation under real-world conditions is limited [188,191,192].

Large-scale microbial innovation in urban, rural, and peri-urban areas requires appropriate policy guidance. Governments should incentivize engineered microbial solutions through performance-based subsidies, carbon and methane offset credits, and financial support for nutrient recovery applications, such as polyhydroxybutyrate (PHB) bioplastics produced by *S. elongatus*, *P. putida*, and *S. elongatus*–*Halomonas boliviensis* consortia [193]. These mechanisms may include carbon credits for biogas generation, certification-based eco-labeling for biosludge reuse, and innovation grants for rural sanitation startups that implement synthetic or precision microbial consortium interventions [5]. Integrated policy–biotechnology models are essential for the transition of synthetic microbial systems from research prototypes to safe, scalable, and socially beneficial WWT solutions.

## 7. Conclusions

Harnessing microbial consortia for next-generation WWTS requires a system-level understanding of the microbial ecology, functional redundancy, and interspecies interactions. This review highlights the critical roles of syntrophic networks, biofilm dynamics, and DIET in sustaining the bioremediation efficiency under dynamic conditions. Technological interventions, such as co-digestion, bioaugmentation, and BESs, have demonstrated significant improvements by enhancing microbial interactions, improving redox stability, and facilitating energy recovery. The integration of multi-omics platforms such as metagenomics, transcriptomics, proteomics, and metabolomics enables high-resolution insights into microbial composition, metabolic pathways, and process resilience. When integrated with AI-driven diagnostics, biosensor platforms, and digital twins, these tools facilitate real-time microbial monitoring, predictive analytics, and early warning systems for operational inefficiencies and the propagation of ARGs. However, certain challenges remain in scaling omics-mediated bioprocesses, particularly in decentralized and resource-limited settings. However, limitations, such as microbial community instability, lack of reproducibility across bioreactor designs, and inadequate real-time monitoring infrastructure, must be systematically addressed. These constraints can be overcome by integrating engineered microbial consortia, intelligent biosensor systems, and ML algorithms into modular, smart bioreactors. Future WWTS are expected to evolve into intelligent and adaptive bioreactors that combine ecological precision and digital optimization. Such systems facilitate the development of circular economic models, advanced climate resilience, and One Health Goals. Priority should be given to field-deployable consortia and biosensors specifically optimized for regionally prevalent xenobiotics, including pesticides, industrial solvents, and pharmaceutical residues. By integrating microbial ecology, synthetic biology, and AI technologies, wastewater management can be reimagined as a globally scalable, sustainable, and bio-intelligent solution for addressing emerging environmental and public health challenges.

## Figures and Tables

**Table 1:** Microbial taxa, functional genes, and tools involved in the biodegradation of major contaminants in WWTS.

Contaminant Type	Key Microbial Taxa/Genera	Major Degradation Genes/Pathways	Analytical/Computational Tools Used	Reference
Pesticides	*Proteobacteria*, *Actinomycetota*, *Thauera*	*opd*, *mpd*, *atzA/B/D/F*, *chd*, *hdx*, *hdl-1*	PICRUSt2	[48,49,50,51]
Hydrocarbons	*Pseudomonas*, *Rhodococcus*, *Betaproteobacteria*, *Sulfuritalea*, *Ottowia*, *Thauera*, *Hyphomicrobium*	Genes involved in benzoate, toluene, and polycyclic aromatic hydrocarbon (PAH) pathways	PICRUSt2, Shotgun Metagenomics	[50,52,53,54]
Endocrine-disrupting compounds/heavy metals	*Proteobacteria*, *Verrucomicrobia*, *Cyanobacteria*	Xenobiotic degradation gene clusters	PICRUSt2	[55,56]
Micropollutants (e.g., pharmaceuticals: diclofenac, venlafaxine)	*Thauera*, *Hyphomicrobium*	Not specifically identified (linked through removal efficiency correlations)	16S rRNA gene sequencing, correlation network analysis	[48,50,57]
Microplastics	*Klebsiella*, *Pseudomonas*, *Sphingomonas*, *Xanthomonas*, *Acinetobacter*	Plastic-degrading enzymes (e.g., PETase, esterases)	16S rRNA gene sequencing	[49]
Surfactants	*Aeromonas*, sulfur-metabolizing bacteria	Genes involved in carbohydrate and sulfur metabolism	Comparative genomics	[54]
Nutrients	Diverse taxa including immobilized microbial consortia	Nitrogen and phosphorus cycling genes (e.g., *amoA*, *nirK*, *phoX*)	Cell immobilization studies, functional genomics	[51]
Pathogenic microbial agents	*Acinetobacter*, total coliforms, fecal coliforms	Not applicable	Activated sludge systems, stabilization ponds	[52]
Dissolved organic matter	Community hubs: rapid-growing taxa; peripherals: slow-growing recalcitrant degraders	Genes for redox reactions, C–N and C–S bond transformation pathways	Comparative genomics, reactomics	[58]

## Data Availability

No new data were created or analyzed in this study.
